# Reimagining Global Health: Accelerating Change for a Sustainable Future

**DOI:** 10.5334/aogh.4616

**Published:** 2025-01-25

**Authors:** Melissa Salm, Ala Alwan, Maureen Lichtveld, Patricia J. García, Peter H. Kilmarx, Nelson K. Sewankambo, Rebecca Martin, Tahmeed Ahmed, Judith N. Wasserheit

**Affiliations:** 1 Department of Anthropology, University of North Carolina Chapel Hill, 207 E. Cameron Ave., Chapel Hill, NC 27599 USA; 2 Director Emeritus, WHO Eastern Mediterranean Region, Cairo 11371, Egypt; 3 School of Public Health, University of Pittsburgh, 130 De Soto St., Pittsburgh, PA 15261 USA; 4 School of Public Health, Universidad Peruana Cayetano Heredia, Av. Honorio Delgado 430, Urb Ingeniería, Lima, Peru; 5 Fogarty International Center, U.S. National Institutes of Health, 31 Center Drive, MSC 2220, Bethesda, MD 20892 USA; 6 Makerere University College of Health Sciences, P.O. Box 7072, Kampala, Uganda; 7 Emory Global Health Institute, 1599 Clifton Road, NE, Atlanta, GA 30322 USA; 8 ICDDR,B, 68 Shaheed Tajuddin Ahmed Sarani, Mohakhali, Dhaka 1212, Bangladesh; 9 Department of Global Health, University of Washington School of Public Health, 3980 15th Ave. NE, Seattle, WA 98105 USA

**Keywords:** Reimagining global health, equity, governance, research, education, sustainable future

## Abstract

In 2023, an interdisciplinary group of global health experts from five continents convened a plenary panel at the Consortium of Universities for Global Health (CUGH) annual conference entitled “Reimagining Global Health for the 21st Century.” At the heart of this viewpoint article lies a fundamental question: How can we reimagine global health to best confront existing challenges and meet the demands of the future? To fully assess the scope of global health challenges and identify sustainable solutions, a clear definition of the aims and strategic approaches is needed. Such an assessment is also critical for progress in promoting equity, including decolonizing global health. Key issues examined are the following: equity, governance, research, education, and sustainability. To assure a sustainable global health enterprise, we propose the following three strategic imperatives as guiding principles: a holistic, unified approach grounded in mutual benefit, joint investment, long‑term collaborative commitment, and solidarity across low‑ to middle‑income countries (LMICs) and high‑income countries (HICs); joint priority setting of investments in research, education, practice, and workforce development; and collaborative governance, maximizing a multisectoral approach among local, national, regional, and global strategies. The viewpoint sets the stage for the development of an action roadmap to develop a unified concept of global health; identify strategies to ensure sustained funding for global health research, education and training, and practice; establish benchmarks and metrics to measure progress; and design a collaborative governance system to promote interconnectedness and engagement among local, regional, national, and global stakeholders.

## Introduction

In 2023, an interdisciplinary group of global health experts from five continents convened a plenary panel at the Consortium of Universities for Global Health (CUGH) annual conference entitled “Reimagining Global Health for the 21st Century.” The discussion focused on various aspects and conceptualizations of global health, as well as how the field should transform in light of emerging challenges, technological developments, governance shifts, and new framings of what constitutes a global health issue. Through subsequent discussions that culminated in this viewpoint, the authors, including the CUGH panelists and CUGH board members, aim to raise awareness of the urgent need to re‑examine and reimagine global health and catalyze broader analyses and discourse across the global health community and its partners and stakeholders. At the heart of external pressures and internal reckonings lies a fundamental question: How can we reimagine global health to best confront existing challenges and meet the demands of the future?

The term “global health” emerged at the turn of the 21st century, largely reflecting perspectives of the Global North [1]. In many countries, particularly low‑ and middle‑income countries (LMICs), the distinction between “global health” and “health” is blurred. Many global health leaders in LMICs have noted that what colleagues in the North call “global health” is what they work on every day as “health [2].” In LMICs such as Bangladesh, “health” refers to healthcare, while global health is considered public health. Yet, they acknowledge that there are challenges such as pandemics and the health impacts of climate change that can only be addressed through transnational strategies and collaborations. And there are other health threats that may not be transnational to the same degree but could benefit from global collaborative efforts in research and response.

The global health field is at an inflection point, compelled to reassess its fundamental principles and practices considering impressive advances, converging challenges, and shifting paradigms. During the last two decades, we have seen major advances in malaria, tuberculosis, and human immunodeficiency virus (HIV) prevention and control, along with reductions in extreme poverty and improvements in health worldwide. These gains reflect significant breakthroughs in research, public health, clinical care, laboratory infrastructure, workforce development, and training capacities in multiple countries [3]. At the same time, we grapple with many global health challenges that require more innovative and effective responses delivered more rapidly. To reinforce measures and initiatives by countries and the international community, in 2015, the United Nations General Assembly set global targets for achievement by 2030 as part of the Sustainable Development Goals (SDGs). Now, almost a decade later, “unprecedented challenges continue to influence the global development landscape, with profound implications for global health [4].” It is estimated that less than one‑third of the SDGs health targets are likely to be achieved by 2030. For example, progress to reduce child and maternal mortality is slow, access to essential health services remains weak, and the overall burden of noncommunicable diseases (NCDs) continues to increase [5]. The situation is compounded by the progress lost during the coronavirus disease 2019 (COVID‑19) pandemic and other health‑related challenges such as climate change, environmental degradation, food insecurity, humanitarian crises, and emergencies, which are likely to increase an already massive resource shortfall, as well as rising trends of societal distrust in governments, evidence‑based health programs and policies, and elevation of leadership that is disdainful of scientific expertise [6, 7]. Simultaneously, powerful movements to decolonize global health have sparked long‑overdue examinations of entrenched inequities and power asymmetries that continue to shape knowledge production and implementation, funding flows, capacity‑building efforts, and governance structures among higher‑ and lower‑income countries [8]. Furthermore, the role and ethics of AI in global health research, education and practice has not been fully examined to date [9, 10].

To fully assess the scope of global health challenges and identify sustainable solutions, a clear definition of the aims and strategic approaches is needed. Such an assessment is also critical for progress in promoting equity, including decolonizing global health. In the absence of a clear definition, it is difficult to reach consensus on priority health challenges that require transnational and global action and on how diverse stakeholders will cooperate and work jointly to address them. Countries across continents, irrespective of socioeconomic level of development, are interconnected and interdependent. Therefore, international cooperation, including sustained and expanded global financial aid, is essential in combating major health risks, and reinforcing health security. A clear concept and interpretation of global health is a cornerstone in achieving this cooperation.

## Key Issues

### Reimagining equity in global health

Koplan et al. [11] defined global health as an area of study, research, and practice that places a priority on improving health and achieving equity in health for all people worldwide, with emphasis on transnational health issues, determinants, and solutions. While this definition is broadly supported, focusing on equity is even more pertinent in view of the lessons learned during the COVID‑19 pandemic. Similarly, consideration of the perspectives from the Global South and the need for global cooperation and coordination in implementing solutions and public health actions should be important factors informing future directions in global health. The principle of equity is and should remain a core pillar of global health [11]. In the last 35 years, quality and access to healthcare has improved substantially around the world, although extreme geographic differences persist [12]. In addition to persistent inequities in access to health services globally, there is an imbalance in financing, resource allocation, educational opportunities, and decision‑making power between high‑ and lower‑income countries [13]. These asymmetries were glaringly laid bare during the COVID‑19 pandemic, during which several countries across income categories were unable to provide adequate hospital capacity, basic supplies, and diagnostics [14], and equitable access to vaccines was undermined by vaccine nationalism, isolationist policies, and corporate pharmaceutical profiteering [15]. While many individuals and laboratories facilitated data‑sharing and genomic analyses in the spirit of global cooperation, the resulting global health tools and products remained inaccessible to many populations in LMICs, which not only reinforced systemic inequities but also undermined the spirit of global solidarity that should have guided global response during a time of a major emergency and may have facilitated ongoing transmission. In the pandemic‑shaped landscape of 21st‑century global health, any reimagining of global health must address this tension, which has been highlighted yet again in the recent challenges in the Pandemic Treaty negotiations led by the World Health Organization (WHO) particularly related to equitable access to vaccines and treatments.

Recently, there have been widespread calls within the field to “decolonize” global health governance, funding, research, education, and training structures and to interrogate the practices and power dynamics underpinning contemporary global health partnerships more broadly [16–18]. These perspectives – which are hardly monolithic – urge the global health community and stakeholders to acknowledge the lingering impacts of colonialist legacies on global health programs and partnerships, and to engage with the deep structural inequities that drive disease and leave health disparities and asymmetrical power relations intact. However, how to build the global health decolonization agenda and how it differs by region in Africa, Latin America, or Asia remain to be defined.

### Reimagining governance in global health

To promote a multisectoral approach, a coordinated governance strategy is necessary to ensure that all stakeholders, including the Global Fund; Gavi, the Vaccine Alliance; and United Nations (UN) agencies such as WHO, UN Environment Programme (UNEP), and UN Children’s Fund (UNICEF), as well as philanthropic and donor organizations, who have been playing a growing role, participate as an action collaborative. The leadership of institutions such as WHO has been increasingly challenged by inadequate financing, proliferation of other actors, and competing mandates. While this diversification of stakeholders has the potential for increased support and resources, it has also contributed to fragmentation, lack of coordination, and the sidelining of the WHO’s normative and technical functions. Global resources are also severely strained with the overlapping replenishment of the Global Fund, Gavi, the Pandemic Fund, the US President’s Emergency Plan for Acquired Immune Deficiency Syndrome (AIDS) Relief (PEPFAR), and other initiatives in the context of post‑pandemic fatigue and economic impacts, as well as global security, climate, and debt crises with diminishing support for global cooperation and development funding in key high‑income countries.

One way to strengthen, if not reimagine, global health governance may be by fostering greater coherence among partners, equally privileging the interests of high‑ and lower‑income countries and assuring that funders’ priorities and community needs are aligned. Another way to reimagine global health governance is to establish mechanisms or protocols that facilitate funders being more directly accountable to the communities in which their resources are spent. The COVID‑19 pandemic has also highlighted the need to strengthen regional approaches to confront health challenges, as demonstrated by the African Centers for Disease Control and Prevention (CDC) or the recently discussed Latin America CDC [19]. In particular, voices from the “Global South” are challenging the field to redefine priorities and frameworks in global health governance [20]. How can global health achieve a more equitable and effective balance in investments by national and international funders, priority setting, and international coordination?

### Reimagining research in global health

The landscape of global health research is also being reimagined. Evolving and emerging health threats – ranging from the tsunami of non‑communicable diseases in LMICs to the multifaceted health impacts of the changing climate, pandemics, antimicrobial resistance hampering the control of a wide range of diseases, and other humanitarian emergencies – demand continuous innovation and development of new interventions. Powerful new scientific tools and approaches have been developed in recent years and are increasingly available to researchers worldwide. These include genetic research and genomic medicine [21], the application of data science together with the use of artificial intelligence [22], and the emergence of implementation science [23].

Research and knowledge generation generally have been a focus of efforts to decolonize global health. The concept of “epistemic injustice” calls attention to the lower credibility afforded to marginalized people – whether by those in high‑income countries (HICs) or by dominant groups in LMICs – and the lack of interpretive resources made available to those who are marginalized [24]. Funders of global health research have key roles in promoting equity in global health research. Efforts are underway to level the playing field by building scientific and research administrative capacity in LMICs, simplifying and making funding decisions more equitable, encouraging more equitable partnerships in international research collaborations, and supporting research priority‑setting in LMICs rather than imposed from HICs [25].

Authorship of research articles has been identified as one metric of equity in international research collaborations. Analyses have generally found that researchers from HICs are in the more influential first and last (senior) authorship positions, while LMIC authors are “stuck in the middle [26].” “Reciprocal innovation” is a key concept for truly equitable partnerships with bidirectional exchange of ideas across diverse global settings [27].

There are promising trends with increasing research capacity in many LMICs and substantial investments in research by both middle‑ and low‑income countries, especially in the wake of the COVID‑19 pandemic. But enduring questions remain about how to democratize the global health research ecosystem in the context of a legacy system of inequality and ongoing imbalances in capacities and access to resources for scientific discovery and innovation [28].

### Reimagining education in global health

Global health education, competencies, and training opportunities should also be reimagined and restructured to address the evolving global health landscape. The dominant model of global health education tends to function as the study of research methods and population health programs and policies to improve health in LMICs via HIC institutions, often, but not always in collaboration with LMIC institutions [29]. How could global health education be reimagined for the 21st century? One way to reimagine and restructure global health education may be to reconceptualize global health education and training using a reciprocal innovation framework and investing in bidirectional training programs and knowledge transfer [30]. Another way may be to cultivate greater in‑country expertise to ensure that global health educational curricula and research agendas are aligned with the priorities and realities of local contexts in both HICs and LMICs.

### Reimagining a sustainable future for global health

Reimagining global health requires creating a collaborative action roadmap to counter the most intransigent health threats facing communities around the world. To assure a sustainable global health enterprise, we propose the following three strategic imperatives as guiding principles:

1. A holistic, unified approach grounded in mutual benefit, joint investment, long‑term collaborative commitment, and solidarity across LMICs and HICs.
2. Joint priority setting of investments in research, education, practice, and workforce development.
3. Collaborative governance, maximizing the interconnectedness, and a multisectoral approach among local, national, regional, and global strategies.

Guided by these principles, the global health community must address several key questions in collaboration with partners and stakeholders:

1. How could an actionable, unified, and decolonized concept of global health be designed in ways that are representative of different perspectives and practices among HICs and LMICs?
2. How do we ensure sustained funding for global health research, education and training, and practice while strengthening and broadening the pathways to develop new global health leaders from both LMICs and HICs with the knowledge, skills, and commitment needed to achieve enhanced transnational and intersectoral cooperation?
3. What are the benchmarks and metrics to measure progress as we reimagine global health?
4. How closely aligned should global health priorities be with internationally agreed‑upon health‑ and policy‑related targets such as the SDGs?
5. How do we establish a collaborative governance system to promote interconnectedness among local, regional, national, and global stakeholder engagement?

As a first step in reimagining global health, agreement on these guiding principles and strategic imperatives and addressing these key questions are meant to set the stage for more in‑depth and focused efforts. We call on the global health community to create opportunities for a deliberate dialogue and debate among LMIC and HIC leaders, implementers, researchers, partners, and stakeholders, including non‑governmental and philanthropic organizations, through workshops, webinars, publications, conferences, and a concerted communication and visibility campaign. Now is the time to build on the impressive advances of the last few decades and reimagine global health to accelerate change for a sustainable future of better health around the world.
